# DLQIad: A New Quality-of-Life Questionnaire for Patients with Atopic Dermatitis

**DOI:** 10.3390/jcm15218695

**Published:** 2026-11-07

**Authors:** Ersilia Tolino, Antonio Di Guardo, Umberto Gallo, Nicoletta Bernardini, Ilaria Proietti, Nevena Skroza

**Affiliations:** 1 Unit of Dermatology ‘Daniele Innocenzi’, Fiorini Hospital, 04019 Terracina, Italy; ersiliatolino@gmail.com (E.T.); umbertogallo2@gmail.com (U.G.); nicoletta.bernardini@uniroma1.it (N.B.); ilaria.proietti@uniroma1.it (I.P.); nevena.skroza@uniroma1.it (N.S.); 2 Dermatology Unit “Daniele Innocenzi”, Department of Medical-Surgical Sciences and Biotechnologies, “Sapienza” University of Rome, Polo Pontino, 04100 Latina, Italy; 3 Istituto Dermopatico dell'Immacolata (IDI-IRCCS), Dermatological Research Hospital, 00167 Rome, Italy

**Keywords:** atopic dermatitis, quality of life, DLQI, biologic therapy, dupilumab, EASI, patient-reported outcomes

## Abstract

**Background/Objectives**: Atopic dermatitis (AD) is a chronic inflammatory skin disease that substantially impairs health-related quality of life (HRQoL). The Dermatology Life Quality Index (DLQI) is widely used, but it is a generic dermatology instrument and may not capture the full patient-reported burden of AD. This study developed an exploratory AD-specific questionnaire (DLQIad), evaluated its internal consistency, and compared its responsiveness with that of the DLQI in patients receiving dupilumab. **Methods**: In this real-life, single-centre observational study, 102 adults with moderate-to-severe AD completed the DLQIad and DLQI and underwent Eczema Area and Severity Index (EASI) assessment before and after 16 weeks of dupilumab treatment. Pre–post differences were evaluated using Wilcoxon signed-rank tests. Internal consistency was assessed using Cronbach’s alpha. Responsiveness was quantified using the standardised response mean (SRM) and the standardised mean-change effect size, and paired patient-level bootstrap resampling was used to compare the DLQIad and DLQI. **Results**: Mean EASI decreased from 27.84 to 5.96, mean DLQI from 15.76 to 2.35, and mean DLQIad from 44.24 to 8.19 (all *p* < 0.001). Cronbach’s alpha was 0.945 at baseline and 0.929 at week 16. The DLQIad showed an SRM of 2.31 compared with 1.89 for the DLQI; the paired difference was 0.421 (BCa 95% CI, 0.134–0.750; *p* = 0.009). The corresponding difference in standardised mean-change effect size was 0.362 (BCa 95% CI, −0.014–0.762; *p* = 0.065). Changes in EASI were significantly associated with changes in both patient-reported instruments, without a significant difference between their standardised regression coefficients. **Conclusions**: The DLQIad demonstrated high internal consistency and preliminary evidence of responsiveness in this selected cohort. Its apparent advantage over the DLQI was dependent on the responsiveness metric used. Structural validity, test–retest reliability, interpretability, and score thresholds require evaluation in independent multicentre samples before clinical implementation.

## 1. Introduction

Atopic dermatitis (AD) is a chronic, relapsing inflammatory skin disease characterised by intense pruritus, eczematous lesions, and a fluctuating course alternating between flares and partial or complete remission [1,2]. The pathophysiology of AD is multifactorial and involves epidermal barrier dysfunction, immune dysregulation, genetic susceptibility, and environmental factors [3]. Beyond visible skin involvement, AD has a substantial effect on patients’ daily lives. Persistent itching, sleep disturbance, pain, limitations in work or school activities, embarrassment, social withdrawal, and anxiety can substantially impair health-related quality of life (HRQoL) [4,5]. The Dermatology Life Quality Index (DLQI) is the most widely used dermatology-specific HRQoL instrument in both clinical trials and routine practice [6]. It is concise and well established, but it was designed for dermatological conditions in general rather than specifically for AD. Its six broad domains may therefore provide limited detail on AD features such as persistent itch, sleep disruption, treatment burden, clothing restrictions, painful fissures, and the psychosocial consequences of recurrent visible lesions. In addition, the DLQI includes response options such as “not relevant”, which can complicate interpretation and comparability [7]. Other available patient-reported measures address important but narrower constructs: POEM focuses mainly on symptom frequency, ItchyQuant on itch severity, and PO-SCORAD on patient-reported disease severity [8,9,10]. These instruments are valuable for their intended purposes, but they are not interchangeable with a measure that integrates AD symptoms, functional limitations, and psychosocial consequences from the patient perspective. The rationale for developing the DLQIad was therefore not that all available questionnaires are inadequate, but that an exploratory complementary instrument might provide a more detailed, integrated profile of AD-related patient-reported burden while retaining a short recall period and straightforward scoring. Dupilumab is recommended for patients with moderate-to-severe AD who are candidates for systemic therapy [11]. Clinical response is commonly assessed using the Eczema Area and Severity Index (EASI) [12], whereas patient-reported measures capture effects that clinician-rated severity scores cannot fully represent. The present study aimed to develop the DLQIad and to provide an initial evaluation of its internal consistency, responsiveness to clinical change, and association with EASI change in adults with moderate-to-severe AD treated with dupilumab. Because this was a single-centre exploratory cohort, the analyses were intended to provide preliminary psychometric evidence rather than to establish definitive score interpretation, dimensional structure, or clinical thresholds.

## 2. Materials and Methods

### 2.1. Study Design and Setting

This was a real-life, single-centre, observational study. The study included consecutive patients with a clinical diagnosis of moderate-to-severe atopic dermatitis who were treated with dupilumab according to current approved indications and routine clinical practice. The study was conducted in accordance with the principles of the Declaration of Helsinki. The study protocol was approved by the local ethics committee, the “Lazio 2” Ethics Committee (protocol number 0054579/2022, dated 9 March 2022). All participants provided written informed consent before inclusion. The cohort was restricted to patients with moderate-to-severe AD receiving dupilumab because the pre–post design required a population expected to undergo measurable clinical change during systemic treatment. The intended population for the preliminary evaluation was therefore adults with moderate-to-severe AD who were candidates for biologic therapy; applicability to mild disease, other systemic treatments, paediatric populations, and untreated patients was not assumed.

### 2.2. Patient Selection and Procedures

Patients were eligible for inclusion if they met the following criteria: (i) diagnosis of atopic dermatitis established by a dermatologist; (ii) moderate-to-severe disease requiring systemic biologic therapy; (iii) treatment with dupilumab according to approved indications; (iv) availability of EASI, DLQI, and DLQIad assessments at baseline and follow-up; and (v) age ≥ 18 years. Exclusion criteria were: (i) incomplete baseline or follow-up questionnaire data; (ii) concomitant dermatological or systemic diseases judged by the investigator to substantially affect HRQoL independently of atopic dermatitis; (iii) major treatment changes during the observation period other than the prescribed dupilumab therapy; and (iv) inability to understand or complete the questionnaires.

A total of 102 consecutive eligible patients were included. Baseline demographic and clinical data were collected, including age, sex, and baseline disease severity. Disease severity was assessed using EASI. HRQoL was assessed using the conventional DLQI and the newly developed DLQIad. Assessments were performed at two time points: baseline, before the initiation of dupilumab therapy, and follow-up, after 16 weeks of treatment, with an allowed time window of ±2 weeks. No a priori sample-size calculation was performed. The analytic sample corresponded to all consecutive eligible patients observed during the enrolment period from January 2023 to December 2025, with follow-up data collected up to April 2026. Therefore, the study should be considered a convenience-sample, exploratory analysis designed to provide preliminary evidence rather than a definitive validation study.

The study population should be distinguished from the eventual scope of the questionnaire. Although the item content was designed to address AD-related patient-reported burden broadly, the present psychometric findings apply only to the moderate-to-severe, dupilumab-treated adults who were studied. Broader use of the DLQIad will require external validation in populations with different disease severity, ages, and treatment pathways.

### 2.3. Development of the DLQIad Questionnaire

The DLQIad was developed as an exploratory AD-specific patient-reported outcome measure intended to complement, rather than replace, the conventional DLQI. The intended construct was AD-related patient-reported burden over the previous 7 days, encompassing three provisional content areas: physical symptoms, functional limitations, and psychosocial impact. Symptom items were included because itching, sleep disturbance, pain, burning, dryness, fissures, and skin sensitivity can directly disrupt daily functioning and emotional well-being. These items were therefore treated as components of the broader burden construct, not as interchangeable measures of HRQoL by themselves. Item generation was informed by common AD manifestations encountered in clinical practice and by the domains covered by existing dermatology and AD measures. An initial pool was reviewed for clarity and relevance by a multidisciplinary clinical team. Fifteen patients with AD then reviewed the wording and perceived relevance of the items, and a 32-patient pilot administration was used to identify unclear or redundant wording. These activities provided informal face-validity input; no formal qualitative concept-elicitation study, saturation analysis, or cognitive-interview framework was performed. Patient comments led to wording simplification and retention of 18 items. To ensure alignment between item stems and response options, every item was phrased as a frequency question and used the same five-category response scale: 0 = Never, 1 = Rarely, 2 = Sometimes, 3 = Often, and 4 = Always. The total score ranges from 0 to 72, with higher values indicating greater AD-related patient-reported burden. Because score bands and a meaningful-change threshold have not yet been established using formal validation and anchor-based methods, no severity categories or minimal clinically important difference were prespecified. The complete version of the DLQIad questionnaire, including all items and response options, is provided in Appendix A.

### 2.4. Statistical Analysis

Continuous variables were summarised using the mean, median, and standard deviation, while categorical variables were reported as frequencies and percentages. Change scores were calculated as post-treatment minus pre-treatment values. Normality of the change scores was assessed using the Shapiro–Wilk test and inspection of their distributions. Because the EASI, DLQI, and DLQIad change scores deviated from normality, within-patient pre–post comparisons were performed using two-sided Wilcoxon signed-rank tests. Two multiple linear regression models were fitted to examine the association between change in clinical severity (ΔEASI) and change in each patient-reported instrument (ΔDLQI and ΔDLQIad), adjusting for age and sex, with female sex as the reference category. To formally compare the strength of the associations, a paired patient-level bootstrap analysis with 10,000 resamples was performed. In each bootstrap replicate, patients were sampled with replacement, both adjusted regression models were refitted, and the difference between the standardised ΔEASI coefficients for DLQIad and DLQI (βDLQIad − βDLQI) was calculated. A bias-corrected and accelerated 95% confidence interval and a two-sided bootstrap *p* value were obtained for this difference. All analyses were performed using R version 4.0.2. Two-sided *p* values < 0.05 were considered statistically significant.

#### 2.4.1. Internal Consistency Assessment

Internal consistency of the 18-item DLQIad was assessed separately at baseline and after 16 weeks using Cronbach’s alpha. Ninety-five percent confidence intervals were calculated using 50,000 non-parametric percentile bootstrap resamples. Corrected item–total correlations and Cronbach’s alpha after deletion of each individual item were also examined. Internal consistency was evaluated for the overall 18-item score; however, Cronbach’s alpha was not interpreted as evidence of unidimensionality or structural validity.

#### 2.4.2. Comparative Responsiveness

Responsiveness was summarised for each instrument using two standardised indices, expressed as absolute magnitudes so that higher positive values indicated greater responsiveness. The standardised response mean (SRM) was calculated as the absolute mean change divided by the standard deviation of the change scores. The standardised mean-change effect size (\(d_{av}\)) was calculated as the absolute mean change divided by the square root of the average of the pre- and post-treatment variances:

$$d_{av}=\left|mean\;\Delta \right|/\sqrt{\left(SD_{pre}^{2}+SD_{post}^{2}\right)/2}$$

Because the DLQI and DLQIad were completed by the same patients, comparative responsiveness was tested using paired patient-level bootstrap resampling with 10,000 replicates. In each replicate, patients were sampled with replacement and both responsiveness indices were recalculated for both instruments. The DLQIad–DLQI difference was then obtained within the same replicate. Bias-corrected and accelerated 95% confidence intervals and two-sided bootstrap *p* values were reported for the paired differences. Percentage reduction from baseline and change relative to each instrument’s maximum possible score were examined as sensitivity analyses.

## 3. Results

### 3.1. Study Population

The study population comprised 102 adult patients with moderate-to-severe atopic dermatitis. Mean age was approximately 41 years (median: 36 years; range: 18–80 years). Sixty-four patients were female (62.7%) and 38 were male (37.3%). All 102 patients (100.0%) were treated with dupilumab (Table 1).

### 3.2. Pre- and Post-Treatment Scores

Two-sided Wilcoxon signed-rank tests showed significant improvements in all three measures after 16 weeks of dupilumab treatment (*p* < 0.001 for all comparisons). Mean EASI decreased from 27.84 ± 5.89 to 5.96 ± 4.50, mean DLQI from 15.76 ± 6.89 to 2.35 ± 2.48, and mean DLQIad from 44.24 ± 14.75 to 8.19 ± 8.99 (Table 2).

The mean change (Δ = post-treatment − pre-treatment) was negative for all three instruments, confirming a consistent reduction in both clinical severity and patient-reported disease burden. The mean change was −21.88 ± 7.09 points for EASI, −13.41 ± 7.08 points for DLQI, and −36.04 ± 15.57 points for DLQIad (Table 3). In raw points, the mean change was numerically larger for the DLQIad than for the DLQI, but the two values are not directly comparable because the instruments have different score ranges. Comparative responsiveness was therefore assessed using standardised indices and paired bootstrap testing in Section 3.5.

### 3.3. Association Between Clinical Severity Change and Patient-Reported Change

Two multiple linear regression models were fitted to assess the association between ΔEASI and the change in each patient-reported measure, adjusting for age and sex.

Model 1–Dependent variable: ΔDLQI = ΔEASI + age + sex. The model was statistically significant overall (R = 0.411; R^2^ = 0.169; adjusted R^2^ = 0.144; SE = 6.553; *p* = 0.00039), explaining 16.9% of the variance in ΔDLQI. ΔEASI was significantly associated with ΔDLQI (B = 0.330; SE = 0.105; β = 0.331; t = 3.155; *p* = 0.002): for each 1-point change in EASI, DLQI changed on average by 0.33 points in the same direction, holding age and sex constant. Sex was a significant covariate, with men showing on average 6.5 points less improvement in DLQI than women, independently of ΔEASI and age (B = 6.538; SE = 1.531; t = 4.269; *p* < 0.001). Age was not significantly associated with ΔDLQI (B = −0.041; *p* = 0.289).

Model 2–Dependent variable: ΔDLQIad = ΔEASI + age + sex. This model was statistically significant overall and explained a comparable proportion of variance (R = 0.387; R^2^ = 0.150; adjusted R^2^ = 0.124; SE = 14.572; *p* = 0.0011). ΔEASI was significantly associated with ΔDLQIad (B = 0.830; SE = 0.233; β = 0.378; t = 3.562; *p* < 0.001). Age was not significantly associated with ΔDLQIad (B = 0.078; *p* = 0.370), whereas male sex was associated with less improvement in DLQIad compared with female sex (B = 9.718; SE = 3.405; t = 2.854; *p* = 0.005) (Table 4).

The standardised coefficient for the association with ΔEASI was numerically higher for the DLQIad than for the DLQI (β = 0.378 versus β = 0.331). However, the formal paired bootstrap comparison showed a difference of 0.047 (BCa 95% bootstrap CI: −0.116 to 0.182; *p* = 0.531). Therefore, the observed numerical difference did not provide statistical evidence that changes in DLQIad were more strongly associated with EASI changes than changes in DLQI.

### 3.4. Standardised and Paired Comparison of Responsiveness

The standardised responsiveness indices for each instrument are shown in Table 5. The DLQIad had an SRM of 2.31 compared with 1.89 for the DLQI, corresponding to an observed paired difference of 0.42. The standardised mean-change effect size was 2.95 for the DLQIad and 2.59 for the DLQI, corresponding to an observed paired difference of 0.36. For comparison, EASI showed an SRM of 3.09 and a standardised mean-change effect size of 4.17.

In the prespecified paired patient-level bootstrap analysis with 10,000 resamples, the DLQIad–DLQI difference in SRM was 0.421 (BCa 95% bootstrap CI: 0.134–0.750; two-sided bootstrap *p* = 0.009) (Table 6). Thus, the DLQIad showed statistically greater responsiveness when responsiveness was quantified using the SRM. The corresponding difference in standardised mean-change effect size was 0.362 (BCa 95% bootstrap CI: −0.014–0.762; *p* = 0.065). Because the confidence interval included zero, the difference between the instruments was not statistically significant according to this second responsiveness index.

As a sensitivity analysis, both instruments were also expressed as the percentage reduction from baseline and as the change relative to each instrument’s maximum possible score. The percentage reduction from baseline was 85.1% for the DLQI and 81.5% for the DLQIad, whereas the change expressed relative to the maximum possible score was 44.7 percentage points for the DLQI and 50.1 percentage points for the DLQIad.

### 3.5. Internal Consistency

Complete item-level data were available for all 102 patients at both assessment time points. The 18-item DLQIad demonstrated high internal consistency at baseline (Cronbach’s α = 0.945; 95% bootstrap CI, 0.927–0.957) and after 16 weeks of treatment (Cronbach’s α = 0.929; 95% bootstrap CI, 0.873–0.952). Corrected item–total correlations ranged from 0.565 to 0.815 at baseline and from 0.530 to 0.756 at week 16. Removal of any individual item resulted in a lower Cronbach’s alpha at both assessment time points, indicating that no single item adversely affected the internal consistency of the scale. Alpha-if-item-deleted values ranged from 0.939 to 0.944 at baseline and from 0.922 to 0.927 at week 16.

## 4. Discussion

This real-life observational study describes the development of the DLQIad, an exploratory AD-specific patient-reported outcome measure, and provides preliminary evidence regarding its internal consistency and responsiveness in 102 adults with moderate-to-severe AD treated with dupilumab. Substantial pre–post reductions were observed in EASI, DLQI, and DLQIad. The reductions in EASI and DLQI observed in our cohort are consistent with the clinical efficacy and improvements in patient-reported quality of life demonstrated for dupilumab in randomised phase 3 trials [13,14]. The DLQI remains the most widely used dermatology-specific HRQoL instrument. It is widely accepted in clinical trials and routine dermatology practice. Its minimal clinically important difference has been estimated at 3.3 points in inflammatory skin diseases, with a recommended practical MCID of approximately 4 points. In addition, the DLQI has validated severity bands and a large body of international experience [14]. Nevertheless, it is not AD-specific. Previous evaluations have highlighted that it focuses more on physical and functional limitations than on psychological dimensions, and that responses may be influenced by age, sex, diagnosis and nationality.

Among AD-specific patient-reported instruments, the Patient-Oriented Eczema Measure (POEM) is one of the best validated and most widely recommended tools. POEM is a 7-item symptom instrument with a score range of 0–28 and can usually be completed in approximately 1 min [8]. In a responsiveness study using data from three randomized trials, the area under the ROC curve was 0.67 for POEM, and the MCID was estimated at 3.4 points. In a validation study of 291 adults with AD, POEM showed better measurement properties than PO-SCORAD, with a meaningful change threshold of −5.0 points and a median completion time of 1 min [15]. These data make POEM a strong comparator for future DLQIad validation, particularly because POEM measures symptoms rather than quality of life.

RECAP and ADCT are also important comparators, but they measure perceived disease control rather than HRQoL. RECAP is a 7-item patient-reported instrument for eczema control, scored from 0 to 28. In a prospective validation study of 200 adults with AD, 82% of a priori hypotheses for single-score validity and 59% for responsiveness were confirmed. Reliability was excellent, with SEMagreement of 1.17 and ICCagreement of 0.988 [16]. The study proposed score bands of 0–1 for completely controlled disease, 2–5 mostly controlled, 6–11 moderately controlled, 12–19 a little controlled and 20–28 not at all controlled. A cut-off of ≥6 was proposed to identify patients whose AD is not under control. The smallest detectable change was 3.2 points, and the minimally important change estimated by predictive modelling was 3.9 points; therefore, an improvement of ≥4 points was proposed as clinically important. Compared with RECAP, the DLQIad is not intended to classify disease control, but to describe how residual AD burden affects daily life. This distinction could be clinically useful in patients whose disease appears “controlled” but who still report sleep impairment, anxiety, embarrassment, intimacy problems or treatment burden.

The ADCT is another validated tool for patient-perceived AD control. In the original longitudinal validation in biologic-treated patients, the ADCT showed strong reliability, with test–retest ICC values of 0.82, 0.78 and 0.79 across month pairs in patients with unchanged global disease assessment. Construct validity was supported by correlations between ADCT and DLQI total score ranging from 0.543 at baseline to 0.846 at month 3, and correlations with patient global assessment ranging from 0.489 to 0.762 [17]. More recent Dutch validation data in 196 adults reported SEMagreement of 1.15 and ICCagreement of 0.983; 82% of single-score validity hypotheses and 59% of responsiveness hypotheses were confirmed [18]. These results show that ADCT and RECAP are psychometrically mature instruments for disease control, while the DLQIad still requires comparable validation before it can be placed at the same evidentiary level.

Against this background, the performance of the DLQIad appears promising but remains preliminary. The questionnaire demonstrated high internal consistency at both assessment time points, with Cronbach’s alpha values of 0.945 at baseline and 0.929 at week 16. All corrected item–total correlations exceeded 0.50, and deletion of any individual item did not increase the overall coefficient. Nevertheless, high internal consistency does not establish the unidimensionality or structural validity of the instrument. The DLQIad showed larger point estimates than the DLQI for both responsiveness indices. The paired difference in SRM was statistically significant (difference = 0.421; BCa 95% CI, 0.134–0.750; *p* = 0.009), whereas the difference in standardised mean-change effect size was not (difference = 0.362; BCa 95% CI, −0.014–0.762; *p* = 0.065). Evidence of greater comparative responsiveness was therefore dependent on the metric used. Changes in EASI were significantly associated with changes in both instruments. Although the standardised ΔEASI coefficient was numerically higher for the DLQIad than for the DLQI, the paired bootstrap difference was not statistically significant (difference = 0.047; BCa 95% CI, −0.116 to 0.182; *p* = 0.531).

If validated, the instrument could also be useful in observational registries and digital monitoring pathways. Repeated DLQIad assessment might help clinicians track whether residual burden shifts over time from physical symptoms to psychosocial or functional consequences. Nevertheless, the preliminary factor solution must first be replicated, and longitudinal measurement invariance should be demonstrated before domain trajectories are interpreted.

### 4.1. Strengths and Limitations

Several limitations must be acknowledged. This was a single-centre exploratory study using a convenience sample, and no a priori sample-size calculation was performed. The dimensional structure of the DLQIad was not evaluated; consequently, the high Cronbach’s alpha coefficients should not be interpreted as evidence of unidimensionality or as definitive support for combining all 18 items into a single score. The item-development process included clinician review, patient wording and relevance review, and a pilot administration, but it did not include formal qualitative concept elicitation, cognitive interviewing, or saturation analysis. The study population was restricted to adults with moderate-to-severe AD receiving dupilumab; generalisability to mild disease, paediatric patients, other treatments, and untreated populations is unknown. Test–retest reliability, measurement error, construct validity against other AD-specific measures, measurement invariance, and cross-cultural validity were not assessed. Finally, no anchor-based meaningful-change threshold or validated score categories were established.

### 4.2. Future Directions

Future studies should be designed as formal multicentre validation studies. The dimensional structure of the DLQIad should first be explored in a sufficiently large sample and subsequently confirmed in an independent cohort. Further evaluation should include test–retest reliability, measurement error, convergent and discriminant validity, known-groups validity, and measurement invariance across clinically relevant subgroups. Comparisons with DLQI, POEM, RECAP, ADCT, and itch or pruritus numerical rating scales would help clarify the distinct contribution of the DLQIad. Anchor-based analyses using patient global ratings of change should be used to estimate meaningful within-patient change, while score categories should be derived and externally validated rather than assumed. Responsiveness comparisons should continue to use paired patient-level methods and should be interpreted according to confidence intervals and across multiple responsiveness metrics.

## 5. Conclusions

In this real-life cohort of 102 adults with moderate-to-severe AD treated with dupilumab, the DLQIad demonstrated high internal consistency and marked responsiveness to clinical improvement. Changes in both DLQIad and DLQI were significantly associated with changes in EASI, without evidence of a significant difference between their standardised regression coefficients. The DLQIad showed statistically greater responsiveness than the DLQI when evaluated using the SRM, whereas the difference in standardised mean-change effect size was not statistically significant. Evidence of greater comparative responsiveness was therefore dependent on the metric used. The DLQIad should currently be regarded as an exploratory and complementary instrument; structural validity, test–retest reliability, anchor-based interpretation, and external validation are required before routine clinical implementation.

## Figures and Tables

**Table 1:** Baseline characteristics of the study population (*n* = 102).

Characteristic	*n* (%) or Value
Age, years	
Mean age	41
Median age	36
Range	18–80
Sex	
Female	64 (62.7)
Male	38 (37.3)
Treatment	
Dupilumab	102 (100.0)

**Table 2:** Descriptive statistics of EASI, DLQI, and DLQIad before and after dupilumab treatment.

Instrument	Mean	Median	SD
DLQIad-pre-treatment	44.24	42.0	14.75
DLQIad-post-treatment	8.19	6.0	8.99
EASI-pre-treatment	27.84	26.0	5.89
EASI-post-treatment	5.96	6.0	4.5
DLQI-pre-treatment	15.76	16.0	6.89
DLQI-post-treatment	2.35	2.0	2.48

**Table 3:** Delta (post − pre) scores for EASI, DLQI, and DLQIad.

ΔScore	Mean	Median	SD
ΔDLQIad	−36.04	−36.0	15.57
ΔEASI	−21.88	−21.0	7.09
ΔDLQI	−13.41	−13.0	7.08

**Table 4:** Multiple linear regression models for ΔDLQI and ΔDLQIad (independent variables: ΔEASI, age, sex; reference sex = female). Change scores were calculated as post-treatment minus pre-treatment values. Female sex was the reference category. Model 1: R^2^ = 0.169, adjusted R^2^ = 0.144, model *p* = 0.00039. Model 2: R^2^ = 0.150, adjusted R^2^ = 0.124, model *p* = 0.0011.

Model/Predictor	B	SE	β (std.)	t	*p*
Model 1: ΔDLQI (R^2^ = 0.169; model *p* = 0.00039)					
Constant	−6.895	2.882	—	−2.392	0.019
ΔEASI	0.330	0.105	0.331	3.155	0.002
Age	−0.041	0.039	−0.100	−1.065	0.289
Sex (male)	6.538	1.531	—	4.269	<0.001
Model 2: ΔDLQIad (R^2^ = 0.150; model *p* = 0.0011)					
Constant	−24.735	6.410	—	−3.859	<0.001
ΔEASI	0.830	0.233	0.378	3.562	<0.001
Age	0.078	0.086	0.086	0.900	0.370
Sex (male)	9.718	3.405	—	2.854	0.005

**Note:** — indicates that the standardized regression coefficient (β) is not applicable to the intercept or was not reported for the categorical predictor.

**Table 5:** Standardised responsiveness indices for EASI, DLQI, and DLQIad.

Instrument	Mean Δ	SD of Δ	SRM	Standardised Mean Change
EASI	−21.88	7.09	3.09	4.17
DLQI	−13.41	7.08	1.89	2.59
DLQIad	−36.04	15.57	2.31	2.95

**Table 6:** Paired patient-level bootstrap comparisons between DLQIad and DLQI.

Comparison (DLQIad − DLQI)	Observed Difference	BCa 95% Bootstrap CI	Two-Sided Bootstrap *p*
Standardised response mean	0.421	0.134 to 0.750	0.009
Standardised mean-change effect size	0.362	−0.014 to 0.762	0.065
Standardised ΔEASI regression coefficient	0.047	−0.116 to 0.182	0.531

## Data Availability

Data is contained within the article.

## Appendix A. The DLQIad Questionnaire

Instructions: For each question, select the response that best describes how often the experience occurred during the past 7 days. The questionnaire should be completed before treatment initiation and after a defined period of treatment. Response scale for every item: 0 = Never; 1 = Rarely; 2 = Sometimes; 3 = Often; 4 = Always. Total score: sum the 18 item scores (range 0–72). Higher scores indicate greater AD-related patient-reported burden. Severity categories and a meaningful-change threshold have not yet been validated and are therefore not provided.

**Table A1:** DLQIad questionnaire items.

#	Item (Past 7 Days)
1	Over the past week, how often did you experience itching?
2	Over the past week, has your sleep been disturbed by itching?
3	Over the past week, have you experienced skin pain or burning?
4	Over the past week, have you noticed bothersome dryness or roughness of the skin?
5	Over the past week, have you had painful fissures or cracks (e.g., hands, skin folds)?
6	Over the past week, have you noticed increased skin sensitivity or irritability (soaps, sweat, heat/cold)?
7	Over the past week, have you had difficulty with personal care (washing, dressing, applying creams)?
8	Over the past week, has your dermatitis limited household, school, or work activities?
9	Over the past week, have you had limitations in physical activity or sport?
10	Over the past week, have you felt restricted in your choice of clothing to avoid irritation or to hide lesions?
11	Over the past week, how often did the time required to apply your treatment cause inconvenience?
12	Over the past week, have you felt embarrassed about the appearance of your skin?
13	Over the past week, have you avoided physical contact (hugs, handshakes) because of your dermatitis?
14	Over the past week, have you had difficulty in social or family relationships?
15	Over the past week, have you experienced discomfort in intimate or sexual life?
16	Over the past week, have you felt anxious or nervous because of your dermatitis?
17	Over the past week, have you felt sad or discouraged about your skin condition?
18	Over the past week, have you had difficulty concentrating or felt irritable because of your dermatitis?
